# Predictors of Severe Coughing and Its Impact on Bronchoalveolar Lavage and Transbronchial Lung Biopsy in Patients with Diffuse Lung Disease: Evaluation of Bronchoscopy Safety

**DOI:** 10.3390/jcm14030893

**Published:** 2025-01-29

**Authors:** Fumi Kobayashi, Takeshi Saraya, Nozomi Kurokawa, Jumpei Aso, Sho Yamada, Kei Nakajima, Kazuyuki Doi, Takatora Akizawa, Ryo Takagi, Narishige Ishikawa, Keisuke Kasuga, Masaoki Saito, Chika Yamaguchi, Hiroki Nunokawa, Yasuo Nakamoto, Manabu Ishida, Mitsuru Sada, Keitaro Nakamoto, Saori Takata, Haruyuki Ishii

**Affiliations:** Department of Respiratory Medicine, Faculty of Medicine, Kyorin University, 6-20-2 Shinkawa, Mitaka City 181-8611, Tokyo, Japan; kobayashi-fumi@ks.kyorin-u.ac.jp (F.K.); conocococco@yahoo.co.jp (N.K.); j_aso@ks.kyorin-u.ac.jp (J.A.); sho616@outlook.jp (S.Y.); kay01431@gmail.com (K.N.); doikazu0428@ks.kyorin-u.ac.jp (K.D.); t_akisan@ks.kyorin-u.ac.jp (T.A.); ryo-m@ks.kyorin-u.ac.jp (R.T.); n-ishikawa@ks.kyorin-u.ac.jp (N.I.); kkclub_0518@ks.kyorin-u.ac.jp (K.K.); treepoint@opal.ocn.ne.jp (M.S.); chika@ks.kyorin-u.ac.jp (C.Y.); hrk910@ks.kyorin-u.ac.jp (H.N.); yasuo-nakamoto@ks.kyorin-u.ac.jp (Y.N.); matsu_manabu@ks.kyorin-u.ac.jp (M.I.); rainbow_orchestra716@yahoo.co.jp (M.S.); keichon2000@yahoo.co.jp (K.N.); s-takata@ks.kyorin-u.ac.jp (S.T.); h141@ks.kyorin-u.ac.jp (H.I.)

**Keywords:** bronchoscopy, BAL, TBLB, cough score, bleeding

## Abstract

**Background/Objectives**: Bronchoscopy is an invasive procedure, and patient coughing during the examination has been reported to cause significant distress. This study aimed to identify predictors of coughing severity and assess its impact on the procedure during bronchoalveolar lavage (BAL) and transbronchial lung biopsy (TBLB). **Methods**: We conducted a prospective study involving 119 consecutive patients with diffuse lung disease who underwent BAL and TBLB at Kyorin University Hospital from April 2019 to December 2023. Cough severity was scored on a scale of 0 to 3, with scores of 0–1 considered mild and 2–3 considered severe. Multivariate logistic regression analysis was performed to identify factors associated with severe coughing during the procedure. **Results**: Severe coughing was significantly associated with Grade 2 or higher bleeding (OR 6.230, 95% CI 2.220–17.400, *p* < 0.001), fewer TBLB specimens collected (OR 0.708, 95% CI 0.530–0.945, *p* = 0.019), and pre-procedural dyspnea (OR 2.560, 95% CI 1.110–5.870, *p* = 0.027). **Conclusions**: Severe coughing during bronchoscopy is associated with increased bleeding and reduced specimen collection. For patients with pre-procedural dyspnea, proactive cough management may improve procedural safety and outcomes.

## 1. Introduction

Bronchoscopy is a crucial procedure in respiratory medicine, recognized for its safety and utility. However, it can be distressing for patients, with coughing during the procedure being a significant factor in reduced patient satisfaction [1]. Adequate sedation improves patient satisfaction, increases acceptance to repeat the procedure, and facilitates the procedure for the operator; therefore, it is recommended unless contraindicated [2,3]. The target level of sedation during bronchoscopy is moderate, with tools like the Ramsay Sedation Scale commonly used for assessment [4]. However, evaluating sedation through commands or stimuli during the procedure is often impractical. Therefore, we focused on assessing cough severity as an easily measurable parameter to better understand its impact on bronchoscopy.

In a prospective survey, we found that patients who experienced significant discomfort during bronchoscopy were more likely to have severe coughing (OR = 1.69, *p* < 0.001), be younger (OR = 0.96, *p* = 0.002), and be examined by less experienced operators (OR = 2.08, *p* = 0.047) [5]. Predictors of severe coughing included endobronchial ultrasound-guided transbronchial needle aspiration (EBUS-TBNA) (OR = 2.95, *p* = 0.004), procedures lasting 36 min or longer (OR = 2.32, *p* = 0.022), and female gender (OR = 2.57, *p* = 0.009).

In a retrospective analysis of endobronchial ultrasound-guided sheath transbronchial biopsy (EBUS-GS-TBB), minimal coughing emerged as a predictor of diagnostic success (OR = 0.455, *p* < 0.001), along with within-lesion findings on radial EBUS (OR = 5.900, *p* < 0.001) and fewer bronchial generations involved (OR = 0.686, *p* < 0.001) [6]. Severe coughing, in contrast, was associated with prolonged procedure duration (OR = 1.030, *p* = 0.045) and the absence of virtual bronchoscopic navigation (OR = 0.449, *p* = 0.017).

Based on these findings, it is clear that coughing during bronchoscopy is a significant factor that can affect both patient discomfort and diagnostic accuracy.

Traditionally, VATS has been considered the gold standard for diagnosing diffuse lung diseases; however, in recent years, the less invasive cryobiopsy has emerged as a more widely adopted approach. Cryobiopsy is a reliable and safe diagnostic method, but its use can be challenging in certain facilities or for specific patients due to the associated risk of bleeding complications [7]. Consequently, TBLB is often employed as an alternative procedure. In contrast, the combination of BAL and TBLB has long been a well-established approach for diagnosing diffuse lung diseases, consistently reported over the decades for its effectiveness and safety. Previous studies have demonstrated diagnostic yields for the combination of BAL and TBLB ranging from 74% to 79.5% [8,9]. Moreover, severe bleeding was observed in only 0.9% of cases [10], underscoring the procedure’s safety and clinical utility, which have supported its widespread use in routine practice.

However, recent research into enhancing the safety of BAL and TBLB has been limited. Addressing this gap, we investigated the impact of coughing during these procedures as a critical factor for improving their safety and effectiveness. Therefore, this study aimed to elucidate the impact of coughing on BAL and TBLB procedures.

## 2. Materials and Methods

### 2.1. Patients

We retrospectively studied consecutive patients who underwent bronchoalveolar lavage (BAL) and transbronchial lung biopsy (TBLB) at the Respiratory Department of Kyorin University Hospital, a 1100-bed tertiary care center in Tokyo, over a period of three years, from April 2019 to December 2023. Patients who underwent other biopsy procedures, such as endobronchial ultrasound-guided sheath transbronchial biopsy (EBUS-GS-TBB), endobronchial ultrasound-guided transbronchial needle aspiration (EBUS-TBNA), and endobronchial biopsy, at the time of BAL and TBLB were excluded from the study.

#### 2.1.1. Bronchoscopy Procedure

Pre-bronchoscopy conferences were held in all cases to identify the purpose of bronchoscopy, the details of the procedure and risk factors such as predisposition to bleeding [11], allergies, and impaired oxygenation prior to the bronchoscopy. All bronchoscopy procedures were performed using flexible bronchoscopes (BF-1TQ290; Olympus, Tokyo, Japan) in an inpatient setting.

Following endotracheal anesthesia and the observation of the airway down to the subsegmental bronchi, the bronchoscope was wedged into the bronchus. BAL was conducted by instilling 50 mL of saline through the bronchoscope channel and then aspirating it while applying negative pressure manually, a process that was repeated three times [12].

Subsequently, under real-time X-ray fluoroscopy guidance, forceps were inserted to the target bronchus. The forceps were positioned 1 cm away from the pleura and closed in sync with the patient’s exhalation. After confirming the absence of chest pain, the forceps were withdrawn. The collected tissue samples were placed in a syringe with saline, subjected to negative pressure, and then fixed in formalin. This TBLB procedure was repeated up to six times when feasible. To prepare for potential bleeding during the biopsy, a diluted solution of 1:100,000 epinephrine was prepared in all cases.

#### 2.1.2. Method of Anesthesia

From April 2019 to July 2020, all patients were anesthetized by the instillation of 5 mL of 2% lidocaine into the throat using a Jackson-type spray (face-to-face application) before bronchoscope insertion. Additional 2% lidocaine, up to a maximum of 20 mL, was administered using the “spray-as-you-go” technique. Lidocaine was sprayed onto the vocal cords before the bronchoscope’s passage through the larynx and subsequently instilled in 1 mL increments from the trachea to the segmental bronchi to ensure adequate local anesthesia [2]. However, due to the SARS-CoV-2 pandemic, the use of pharyngeal and laryngeal anesthesia with the Jackson-type spray was discontinued from August 2020 to December 2023 to mitigate the risk of SARS-CoV-2 exposure to medical staff. During this period, pharyngolaryngeal and bronchial anesthesia were administered using a spray catheter (PW-6C-1, Olympus, Tokyo, Japan) after the scope was inserted into the mouth, with lidocaine applied from the vocal cords to the subsegmental bronchi [13].

Prior to the examination, 1 to 3 mg of midazolam and 35 mg of pethidine were administered intravenously for sedation. An additional 1 mg of midazolam was administered as needed to maintain moderate sedation throughout the procedure [14]. Moderate sedation was defined as preserving spontaneous breathing and responsiveness to verbal stimuli, equivalent to a Ramsay score of 3 to 4. The bronchoscope was introduced through the mouth, and if coughing occurred during the procedure, 2% lidocaine was repeatedly administered through the bronchoscope channel.

#### 2.1.3. Score of Cough Severity on Bronchoscopy

Soon after completion of the bronchoscopy, the bronchoscopist evaluated the severity of coughs during the procedure and divided them into four grades, ranged from scores 0 to 3, which are defined as score 0 (none; no cough), score 1 (mild; cough present but does not interfere with the procedure), score 2 (moderate; cough necessitates temporary interruption within the trachea), and score 3 (severe; cough requires scope removal from the trachea) (Table 1), as in our previous report [5,6]. This evaluation was routinely recorded using a standardized bronchoscopy report template. However, it was based on the bronchoscopist’s subjective judgment.

The predictors of severe cough in BAL and TBLB were evaluated by dividing patients into two groups: with a cough score of 0 or 1, as the mild cough group, and a cough score of 2 or 3, as the severe cough group.

#### 2.1.4. Diagnostic Criteria in Bronchoscopy

The diagnostic validity of bronchoscopy was assessed by evaluating whether the TBLB tissue findings contributed to the final diagnosis. The final diagnosis was determined by the clinician based on the patient’s clinical course, blood test results, imaging findings, and bronchoscopy results such as BAL and TBLB, with additional input from VATS findings when available. It is important to note that BAL findings were not incorporated into the assessment of diagnostic validity in this study. The evaluation of diagnostic validity was conducted using a four-tiered scale.

Findings categorized as “Specific” were those consistent with established guidelines or literature, such as idiopathic pulmonary fibrosis [15], IIPs [16,17], hypersensitivity pneumonitis [18], connective tissue disease associated interstitial lung disease (CTD-ILD) [19], and drug-induced lung injury [20]. Findings labeled as “Probable” were not specific but still aligned with the final diagnosis. “Not Specific” findings were non-contributory and did not aid in establishing a diagnosis. Lastly, cases with an “Insufficient Sample” indicated that the tissue sample was inadequate for a definitive diagnosis (Table 2).

For further analysis, findings categorized as “Specific” and “Probable” were combined into the “Diagnostic” group, while “Not Specific” and “Insufficient Sample” findings were grouped into the “Non-Diagnostic” category [21,22].

#### 2.1.5. Collection of Associated Date

We collected data on patient characteristics such as age, gender, smoking history and index, height, weight, BMI, history of asthma or COPD, and whether the patient was taking antithrombotic drugs that required withdrawal before bronchoscopy. In all such cases, the patients adhered to the prescribed withdrawal period for their antithrombotic drugs [11]. Pre-procedural assessments included symptoms like cough; sputum production; dyspnea; oxygenation impairment; and blood tests for KL-6, CRP, and white blood cell count, as well as the proportions of eosinophils and neutrophils. Imaging findings from chest X-rays and CT scans were also evaluated. Procedural data included the operator’s experience (≥5 years or <5 years), method of pharyngolaryngeal anesthesia (Jackson spray or spray catheter), BAL site (middle lobe and lingular/other), fluoroscopic visibility (visible or invisible), number of TBLB samples (≥4 or <4), procedure duration, and bleeding severity (≥Grade 2 or <Grade 2). The severity of bleeding was determined on a scale of four steps, according to the standardized definitions of bleeding [23]: Grade 1, bleeding requiring less than 1 min of suctioning or wedging of the bronchoscope; Grade 2, bleeding requiring more than 1 min of suctioning or wedging or the instillation of cold saline, diluted vasoactive substances, or thrombin; Grade 3, bleeding requiring selective intubation using an endotracheal tube or balloon/bronchial blocker for less than 20 min; and Grade 4, bleeding requiring persistent selective intubation for more than 20 min, blood transfusion, bronchial artery embolization, or intensive care therapy and transient oxygen supplementation during bronchoscopy (≥10 L/min or <10 L/min). Post-procedural outcomes included TBLB diagnostic success; BALF recovery rate; and complications of pneumothorax, acute exacerbation of interstitial pneumonia, bacterial pneumonia, and fever.

This study was approved by the Ethics Committee of Kyorin University (approval number: 2273).

#### 2.1.6. Statistical Analysis

Continuous variables were expressed as the median and interquartile range (IQR), unless otherwise stated, and were compared using the Mann–Whitney *U* test. Categorical variables were compared using Fisher’s exact test. Multivariable analyses were conducted using multiple logistic regression models. A *p*-value of less than 0.05 was considered statistically significant. All statistical analyses were performed using EZR version 1.40 [24].

## 3. Results

Between March 2019 and December 2023, a total of 1355 bronchoscopy procedures were performed at our respiratory department. Of these, 131 cases were planned for BAL and TBLB in patients with diffuse lung disease. Twelve cases were excluded from the analysis due to missing cough data (*n* = 9), non-performance of BAL (*n* = 2), or non-performance of TBLB (*n* = 1), leaving 119 cases for the final analysis.

### 3.1. Patient Characteristics and Clinical Background

The median age of the study population was 68 years (IQR 55; 75), with 68 male patients (57.1%). The distribution of cough severity among patients was as follows: 9 patients (7.6%) had a cough score of 0, 39 patients (32.8%) had a score of 1, 64 patients (53.8%) had a score of 2, and 7 patients (5.9%) had a score of 3.

The final diagnoses included hypersensitivity pneumonitis in 20 cases, connective tissue disease-associated interstitial lung disease (CTD-ILD) in 19 cases, organizing pneumonia in 16 cases, eosinophilic pneumonia in 12 cases, idiopathic pulmonary fibrosis (IPF) in 7 cases, drug-induced lung injury in 7 cases, and infections in 7 cases. In addition, pulmonary alveolar proteinosis (PAP) was diagnosed in six cases, nonspecific interstitial pneumonia (NSIP) in four cases, unclassifiable interstitial pneumonia in four cases, graft-versus-host disease (GVHD) in three cases, malignancy in two cases, Castleman disease in two cases, and pulmonary infiltration associated with myelodysplastic syndrome (MDS) in two cases. Other diagnoses included radiation pneumonitis, upper-lobe predominant pulmonary fibrosis, alveolar hemorrhage, diffuse osteodysplasia, IgG4-related disease, and sarcoidosis, each in one case. There were six cases corresponding to the aforementioned diagnoses, and in two cases, the diagnosis remained unknown.

**Figure 1:**
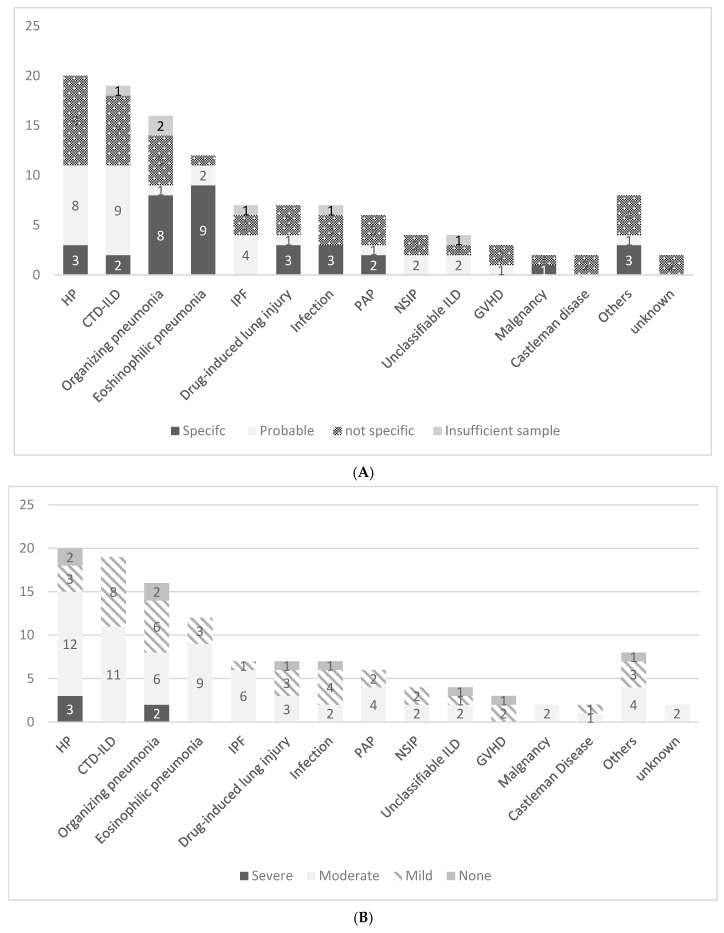
(**A**) Proportion of diagnostic group based on TBLB by disease. (**B**) Proportion of severe cough group by disease.

TBLB histological findings allowed for a diagnosis (specific or probable) in 53.8% of the cases overall. Detailed classifications of diagnostic accuracy are provided in Figure 1A, while the classification of cough score is shown in Figure 1B.

Table 3 shows the differences in diagnostic rates of TBLB and cough severity among 67 cases focused on the four main diseases (HP, CTD-ILD, OP, and EP). Fisher’s exact test revealed that EP had a significantly higher diagnostic success rate compared to the other three diseases (11 [91.7%] vs. 31 [56.3%] *p* = 0.0241). However, there were no significant differences in cough severity across the comparisons.

### 3.2. Comparison Between Mild and Severe Cough Groups

In comparison between the mild and severe cough groups, patients in the severe cough group had a significantly higher median BMI (kg/m^2^) (23.51 [20.30–25.55] vs. 21.24 [19.05–23.13], *p* = 0.016) and a higher prevalence of dyspnea prior to the procedure (45 [63.4%] vs. 21 [43.8%], *p* = 0.040). Additionally, the severe cough group exhibited a lower median neutrophil percentage (%) (67.9 [59.1–72.2] vs. 72.8 [62.3–79.0], *p* = 0.015) and a higher median eosinophil percentage (%) (2.80 [1.70–4.85] vs. 1.6 [0.8–4.4], *p* = 0.015) in the blood. Furthermore, the severe cough group had a higher median KL-6 level (U/mL) (790 [352–1657] vs. 475 [284–835], *p* = 0.008) and a significantly higher proportion of patients with ground-glass opacities on CT (68 [95.8%] vs. 39 [81.2%], p = 0.013). Although the comparison was limited to smokers (n = 58), the smoking index was significantly lower in the severe cough group compared to the mild cough group [22.75 (11.4–38.8) vs. 40.0 (24.0–50.0), *p* = 0.032]. 

The findings during the procedure include that patients in the severe cough group had a lower median number of TBLB specimens collected (4.00 [3.00–5.00] vs. 5.00 [4.00–6.00], *p* = 0.012) and experienced a higher incidence of Grade 2 or greater bleeding during the procedure (32 [45.1%] vs. 6 [12.5%], *p* < 0.001). Although the diagnostic yield of TBLB was lower in the severe cough group than in the mild cough group (34 [47.9%] vs. 30 [62.5%], *p* = 0.136), this difference was not statistically significant. The median recovery rate of BALF (%) was comparable between the two groups (53.3 [47.0–60.9] vs. 54.3 [48.0–63.3], *p* = 0.749) (Table 4).

### 3.3. Multivariate Analysis of the Factors Associated with Severe Cough

Multivariate analysis of the factors found to be significant in univariate analysis identified several variables significantly associated with severe coughing. Specifically, patients who experienced Grade 2 or higher bleeding (the definition is described in Material and Methods) during the procedure were more likely to have severe cough (OR 6.230, 95% CI 2.220–17.400, *p* < 0.001). Additionally, the presence of dyspnea before the procedure was another significant factor (OR 2.560, 95% CI 1.110–5.870, *p* = 0.027). Furthermore, a smaller number of TBLB specimens collected was inversely related to severe cough (OR 0.708, 95% CI 0.530–0.945, *p* = 0.019) (Table 5).

### 3.4. Relationship Between Cough Severity and Bleeding Severity

**Figure 2:**
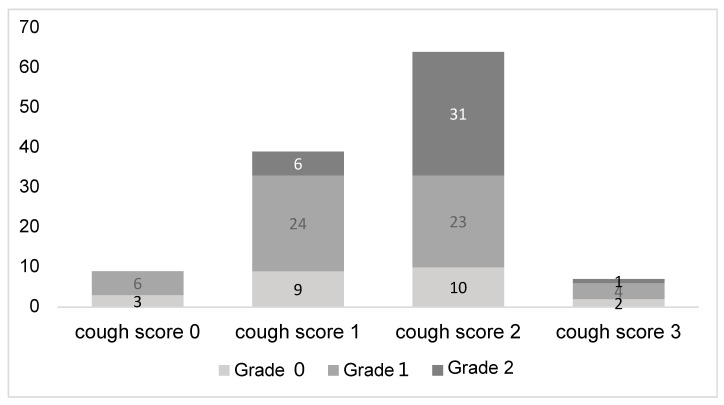
Bleeding severity by cough score. Intra-procedural Grade 2 bleeding increased with higher cough scores, occurring at rates of 0%, 15.4%, and 48.4% for cough scores of 0, 1, and 2, respectively.

Figure 2 illustrates the relationship between cough severity and the severity of bleeding during bronchoscopy. No patients with a cough score of 0 experienced Grade 2 bleeding, while 15.4% of those with a cough score of 1 and 48.4% of those with a cough score of 2 had Grade 2 bleeding. This demonstrates a clear association between increasing cough severity and the likelihood of more severe bleeding during the procedure.

## 4. Discussion

Previous reports have shown that coughs during bronchoscopy can increase patient distress and reduce tolerance for repeat examinations [1,5,25]. Additionally, it has already been reported that severe coughing during EBUS-GS-TBB procedures can lower diagnostic rates [6]. However, no prior studies have specifically examined the impact of coughs in relation to BAL and TBLB procedures. Therefore, this study is the first to identify three key factors associated with severe cough during bronchoscopy using multivariate logistic regression: intra-procedural bleeding (Grade 2 or higher), pre-procedural dyspnea, and a lower number of TBLB specimens collected.

First, Grade 2 or higher bleeding during the procedure was the factor most strongly associated with severe coughing. Although there have been no previous reports on the direct involvement of coughing and bleeding during bronchoscopy, it is often experienced that blood gushes from the peripheral bronchi following severe coughing after TBLB in clinical practice. Additionally, coughing may be triggered by the presence of blood or by stimulation from the administration of vasoconstrictive agents. In this study, determining the exact causal relationship between coughing and bleeding was challenging. However, bleeding during bronchoscopy can sometimes be life-threatening [11], and paying attention to coughing may contribute to safer bronchoscopy procedures.

Next, pre-procedural dyspnea was associated with severe coughing. Dyspnea is reported in 78.16% of patients with interstitial pneumonia prior to bronchoscopy and is considered a common symptom [26]; however, its relationship with coughing during the procedure has not been clarified. Nonetheless, our univariate analysis revealed that findings such as the presence of ground-glass opacities and elevated KL-6 levels—both of which are commonly observed in the acute phase of interstitial pneumonia—were significant. This suggests that dyspnea might similarly indicate the acute phase of interstitial pneumonia. While this study included a variety of diffuse lung diseases, performing bronchoscopy during such an acute phase may be related to the occurrence of severe coughing.

The third point concerns the association between severe coughing and the reduction in the number of TBLB specimens. Previous studies have reported that increasing the number of TBLB specimens improves the diagnostic yield [27]. Therefore, it can be said that obtaining more specimens is desirable for improving diagnostic accuracy. However, when severe coughing makes breath holding difficult during biopsy, it may be challenging to maintain the proper distance between the pleura and forceps, potentially increasing the risk of pneumothorax. This could lead the physician to terminate the procedure early. While coughing did not directly affect the diagnostic yield or pneumothorax in this study, managing coughing may be important to ensure sufficient specimen collection.

Regarding the diagnostic yield of TBLB, specific or consistent histological findings were obtained in 64 cases (53.8%) in this study. Previous studies using similar diagnostic criteria have reported diagnostic rates ranging from 30.4% to 59.1%, which is consistent with our findings [22].

An additional analysis focusing on the four diseases with the highest number of cases (HP, CTD-ILD, OP, and EP) demonstrated that the diagnostic rate for EP was significantly higher compared to the other three diseases (Table 3). Although tissue diagnosis is not considered essential for acute EP, it is regarded as desirable for chronic EP to differentiate it from other conditions [28]. While no standardized histological criteria for eosinophilic pneumonia currently exist, we adopted the presence of interstitial inflammation with eosinophilic infiltration as the specific diagnostic criterion for EP, achieving a diagnostic rate of 91.7% [29].

Previous studies on the diagnostic rates of diffuse lung diseases have not specifically examined EP due to its rarity. However, in this study, EP accounted for a relatively high proportion of cases (10.1%), and the diagnostic rate for EP was shown to be higher compared to other diseases. Although this analysis was limited to four selected diseases and should be interpreted with caution, TBLB was considered to have significant utility for diagnosing EP.

This study had several limitations. Firstly, it was a retrospective, single-center analysis, which may be subject to various biases. However, the study included consecutive cases of bronchoscopy over a period of more than four years, suggesting that there was no selection bias in the included cases. Future studies in a multicenter setting would be desirable to validate these findings. Secondly, regarding the assessment of pre-procedural dyspnea, the presence or absence of dyspnea was recorded retrospectively by reviewing medical records. This approach did not allow for a standardized severity evaluation, such as using the modified Medical Research Council (mMRC) scale, potentially limiting the accuracy of dyspnea assessment. Additionally, moderate bleeding in patients undergoing TBLB is generally reported to occur in 1.1–2.8% of cases; however, in this study, Grade 2 bleeding was observed in 31.9% of cases, a significantly higher result [11]. One possible reason for this is that vasoconstrictors were prepared for use in all cases, resulting in a lower threshold for their use, and cases where vasoconstrictors were used were categorized as Grade 2 [23]. This is supported by the fact that there were no cases of Grade 3 or higher bleeding. However, in cases where vasoconstrictors were used, the bleeding duration and volume were expected to be greater compared to cases where they were not used, so this should not affect the conclusion that severe coughing is related to increased bleeding. Lastly, it is worth noting that pethidine was used for analgesia instead of fentanyl. While fentanyl is generally used as an opioid for its antitussive effects [2,3], due to our institutional restrictions, pethidine was used in all cases, and the data reflect this.

Thus far, despite these limitations, we were the first to demonstrate the relationship between cough severity during bronchoscopy and its potential contributing factors.

## 5. Conclusions

Severe coughing during bronchoscopy was associated with Grade 2 or higher bleeding, pre-procedural dyspnea, and a reduction in the number of specimens collected. In other words, effective cough management, particularly in patients with dyspnea, may help obtain more specimens and ensure a safer procedure with reduced bleeding.

## Figures and Tables

**Table 1:** Cough score.

Cough Score	Description	Impact on Procedure
0	None	No cough present
1	Mild	Cough present but does not interfere with the procedure
2	Moderate	Cough necessitates temporary interruption within the trachea
3	Severe	Cough requires scope removal from the trachea

**Table 2:** Classification of tissue findings in bronchoscopy.

Category	Description
Specific	Findings consistent with guidelines or similar standards
Probable	Nonspecific findings that align with the final diagnosis.
Not Specific	Findings that do not contribute to the diagnosis.
Insufficient Sample	The sample quantity was insufficient for diagnosis

**Table 3:** Comparison of the diagnostic rates of TBLB and cough severity in four major diseases.

	*n*	Diagnostic Utility		Cough Severity	
		Diagnosed Group	Non-Diagnosed Group	Mild Cough Group	Severe Cough Group
HP	20	11 (55.0%)	9 (45.0%)	5 (25.0%)	15 (75.0%)
CTD-ILD	19	11 (57.9%)	8 (42.1%)	8 (42.1%)	11 (57.9%)
OP	16	9 (56.3%)	7 (43.7%)	8 (50.0%)	8 (50.0%)
EP	12	11 (91.7%) *	1 (8.3%)	3 (25.0%)	9 (75.0%)
Total	67	42 (62.7%)	25 (37.3%)	24 (35.8%)	43 (64.2%)

Statistical analysis: * comparison of diagnostic utility between the EP group and other groups, *p* < 0.05.

**Table 4:** Comparison between the mild and severe cough groups.

Variable	Mild Cough Group (*n* = 48)	Severe Cough Group (*n* = 71)	*p* Value
Age, Median [IQR], years	70.5 [58.5–75.0]	65.0 [50.5–75.0]	0.294
Sex			
Male	30 (62.5%)	38 (53.5%)	0.352
Female	18 (37.5%)	33 (46.5%)	
Smoking History			
Yes	25 (52.1%)	33 (46.5%)	0.579
No	23 (47.9%)	38 (53.5%)	
Smoking index, Median [IQR], pack year	40.0 [24.0–50.0] (*n* = 25)	22.75 [11.4–38.8] (*n* = 33)	0.032
BMI, Median [IQR], kg/m^2^	21.24 [19.05–23.13]	23.51 [20.30–25.55]	0.016
Asthma History			
Yes	6 (12.5%)	10 (14.1%)	1.000
No	42 (87.5%)	61 (85.9%)	
Requirement for antithrombotic withdrawal			
Yes	10 (20.8%)	6 (8.5%)	0.061
No	38 (79.2%)	65 (91.5%)	
WBC, Median [IQR],/μL	7400 [5900–9625]	7500 [6000–9000]	1.000
Blood Neutrophils, Median [IQR], %	72.8 [62.3–79.0]	67.9 [59.1–72.2]	0.015
Blood Eosinophils, Median [IQR], %	1.6 [0.8–4.4]	2.80 [1.70–4.85]	0.015
CRP, Median [IQR], mg/dL	2.4 [0.2–5.4]	1.0 [0.2–4.6]	0.431
KL-6, Median [IQR], U/mL	475 [284–835]	790 [352–1657]	0.008
Lung Abnormalities on chest X-ray			
Visible	38 (79.2%)	63 (88.7%)	0.194
Invisible	10 (20.8%)	8 (11.3%)	
Ground-glass Opacity on Thoracic CT			
Yes	39 (81.2%)	68 (95.8%)	0.013
No	9 (18.8%)	3 (4.2%)	
Infiltrative Shadow on Thoracic CT			
Yes	29 (60.4%)	40 (56.3%)	0.708
No	19 (39.6%)	31 (43.7%)	
Emphysematous Change on Thoracic CT			
Yes	10 (20.8%)	14 (19.7%)	1.000
No	38 (79.2%)	57 (80.3%)	
Honeycomb Lung on Thoracic CT			
Yes	3 (6.2%)	6 (8.5%)	0.738
No	45 (93.8%)	65 (91.5%)	
Operator Experience			
≥5 years	15 (31.2%)	24 (33.8%)	0.844
<5 years	33 (68.8%)	47 (66.2%)	
pharyngolaryngeal anesthesia			
Jackson spray	8 (16.7%)	19 (26.8%)	0.265
Spray catheter	40 (83.3%)	52 (73.2%)	
BAL Site			
Middle Lobe or Lingular	32 (66.7%)	54 (76.1%)	0.300
Other	16 (33.3%)	17 (23.9%)	
Number of TBLB Specimens, Median [IQR]	5.00 [4.00–6.00]	4.00 [3.00–5.00]	0.012
Procedure Duration, Median [IQR], min	34.00 [29.00–37.25]	33.00 [29.00–38.00]	0.888
Intra-procedural Bleeding			
Grade 2 or higher	6 (12.5%)	32 (45.1%)	<0.001
Grade 1 or lower	42 (87.5%)	39 (54.9%)	
Oxygen supplementation			
≥10 L/min	3 (6.2%)	11 (15.5%)	0.155
<10 L/min	45 (93.8%)	60 (84.5%)	
TBLB diagnostic success			
Yes	30 (62.5%)	34 (47.9%)	0.136
No	18 (37.5%)	37 (52.1%)	
BALF Recovery Rate, Median [IQR], %	54.3 [48.0–63.3]	53.3 [47.0–60.9]	0.749
Post-procedural Pneumothorax			
Yes	1 (2.1%)	1 (1.4%)	
No	47 (97.9%)	70 (98.6%)	1.000
Post-procedural acute exacerbation of interstitial pneumonia			
Yes	1 (2.1%)	0 (0%)	
No	47 (97.9%)	71 (100%)	0.403
Post-procedural bacterial pneumonia			
Yes	1 (2.1%)	1 (1.4%)	
No	47 (97.9%)	70 (98.6%)	1.000
Post-procedural fever			
Yes	2 (4.2%)	2 (2.8%)	1.000
No	46 (95.8%)	69 (97.2%)	

IQR: Interquartile range.

**Table 5:** Multivariate analysis of the factors associated with severe cough.

Variable	Odds Ratio	95% Confidence Interval	*p*-Value
Intra-procedure Bleeding (Grade 2 or higher)	6.23	2.220–17.400	<0.001
Number of TBLB Specimens	0.708	0.530–0.945	0.019
Pre-procedure Dyspnea	2.56	1.110–5.870	0.027

## Data Availability

The original contributions presented in the study are included in the article, further inquiries can be directed to the corresponding authors.

## Abbreviations

The following abbreviations are used in this manuscript: BAL: Bronchoalveolar lavage, TBLB: Transbronchial lung biopsy, EBUS-TBNA: Endobronchial ultrasound-guided transbronchial needle aspiration, EBUS-GS-TBB: Endobronchial ultrasound-guided sheath transbronchial biopsy, HP: Hypersensitivity pneumonia, CTD-ILD: Connective tissue disease-associated interstitial lung disease, OP: Organizing pneumonia, EP: Eosinophilic pneumonia, IPF: Idiopathic pulmonary fibrosis, NSIP: Nonspecific interstitial pneumonia, Unclassifiable ILD: Unclassifiable interstitial lung disease, PAP: Pulmonary alveolar proteinosis, GVHD: Graft-versus-host disease, MDS: Myelodysplastic syndrome, VATS: Video-assisted thoracoscopic surgery, BMI: Body mass index, and SARS-CoV-2: Severe acute respiratory syndrome coronavirus 2.
